# The great divide between employees: Clustering employee “well-being” during a pandemic

**DOI:** 10.1371/journal.pone.0294540

**Published:** 2025-03-31

**Authors:** Jacques Bughin, Michele Cincera, Dorota Reykowska, Marcin Żyszkiewicz, Rafal Ohme

**Affiliations:** 1 Université libre de Bruxelles, Solvay Brussels School of Economics and Management, iCite and ECARES, Brussels, Belgium; 2 MachaonAdvisory, Brussels, FortinoCapital, Brussels, Antler, Amsterdam and Portulans Institute, Washington, United States of America; 3 Neurohm, dWSB University, Torun, Poland; CNR: Consiglio Nazionale delle Ricerche, ITALY

## Abstract

The Covid-19 pandemic is a textbook case of significant situational stress induced by various disruptions beyond mere health concerns, such as social isolation and financial constraints. For the workforce, it is essential to anticipate how these disruptions may undermine employees’ resilience, to avoid a negative spiral where poor well-being lowers productivity, reduces economic prospects, and continues to increase worker stress. We measure multiple forms of stress and worries as drivers of well-being—health, economic, social, and psychological—encountered by the workforce during the acute period of the Covid-19 pandemic. The study analyzed data from 2,780 employees across five European countries: France, Germany, Italy, Spain, and Sweden. Overall Concern Score: The overall concern score was 56.8% across four domains: health, economic, social, and psychological. Stressors can be synthesized into five typical groups associated with a variety of mediating factors such as institutional trust, lifestyle, and worker education. The implication is that workers’ well-being is heterogeneous and that human resource practices may need at least a segmented approach to well-being if they wish to create an environment of a resilient and productive workforce.

## Introduction

According to the Worldometers database, the Covid-19 pandemic had officially affected more than 700 million people worldwide by May 2024, causing around 7 million deaths. This makes it one of the most challenging pandemics to manage since the Spanish flu at the beginning of the 20th century [1].

The impact of such a pandemic is recognized not only on health but also on economic livelihoods. At the height of the pandemic, the WHO, FAO, and ILO issued a joint statement reflecting the negative fallout in other areas, claiming that “tens of millions of people were at risk of falling into extreme poverty and millions of businesses faced an existential threat” [2] demonstrated a significant peak in small businesses, but beyond small entrepreneurs, a large part of the workforce is also employed in large corporations [3,4], and if bankruptcy became more limited for large firms, the workforce during the crisis was not faring well [5]. Stress particularly increased in most exposed professions, such as medical staff, teachers, or security forces, but it spread to the workforce as a whole, with a 70% rise in burn-out during the peak of the pandemic in the US in 2020 [6,7]. Workforce well-being is strongly associated with increased labor productivity [8]. Failure to promptly address well-being may lead to an economic depression, which feeds a negative spiral of ill-being and poor economic prospects, propelling the health crisis into a lasting economic crisis. Many companies have tried implementing preventive health measures against Covid-19, including health protection measures in the workplace or working from home. However, these health measures may lack scope (only around 40% of workers, and especially managers, could work from home; see [9,10] and may miss out on a range of additional concerns, such as job retention and finances, or the ability to protect close ties [11].

This study is grounded in the Conservation of Resources (COR) theory, developed by [12]. COR theory posits that individuals strive to obtain, retain, and protect their resources, which include objects, personal characteristics, conditions, and energies. When these resources are threatened or lost, individuals experience stress. The pandemic has created a significant threat to various resources such as health, economic stability, and social connections, which are critical for employee well-being.

This article is part of a recent stream of wellness science literature investigating the different dimensions of pandemic-related stress beyond pure health worries. [13] examine the combined role of economic concerns, social distancing, and health anxieties in anticipating the possibility of significant mental health developments from the pandemic. [14] consider economic anxiety to be as big a problem as health anxiety in countries such as the USA, the UK, and Israel.

The study aligns with the concept of well-being, rooted in positive psychology, rather than wellness in the context of medicine. Well-being in positive psychology focuses on the holistic experience of life satisfaction and the balance of positive and negative emotions [15]. It encompasses various domains such as emotional, social, and psychological well-being [16]. This framework is appropriate for understanding the diverse impacts of the Covid-19 pandemic on employees, considering their mental and emotional health, social interactions, and economic stability.

The primary research problem addressed in this study is understanding how the multifaceted disruptions caused by the Covid-19 pandemic impact the well-being of employees, and how these impacts can be segmented to inform targeted human resource practices. The objectives of this research are first, to measure the various forms of stress and worries—health, economic, social, and psychological—encountered by the workforce during the acute period of the Covid-19 pandemic. Second, to identify and cluster the typical groups of stressors and mediating factors such as institutional trust, lifestyle, and worker education. Third, to analyze the relationship between these clusters and employee socio-demographic characteristics, protective behaviors, and levels of social trust. Fourth, to examine the differences in well-being concerns across five European countries and how these are influenced by each country’s social and economic fabric. And finally, to utilize online response time as an indicator of attitude strength to adjust survey responses and reinforce the statistical significance of the findings.

To address these objectives several research questions are posed. What are the predominant types of stress and worries affecting employees during the Covid-19 pandemic? How can these stressors be clustered into distinct groups? What socio-demographic characteristics, behaviors, and levels of trust are associated with each cluster of stressors? How do these clusters and their associated characteristics vary across different European countries? And how does response time adjustment affect the reliability of the survey responses regarding employee worries?

The contribution of this research is fivefold. Firstly, to our knowledge, the research is one of the first to study a wide range of concerns perceived by the workforce. The risks assessed include occupational and financial risks (micro-economic risks), risks related to the satisfaction of basic needs (supply chain), violence, and psychological risks (social risks), and the country’s finances (macro-economic risks), in addition to health-related risks. The first finding is that health accounts for just over 40% of all risks expressed by workers.

Secondly, worries can be clustered. Five polarized segments of the working population seem to prevail regarding perceived worries encountered. An important study by [17] also uses clustering techniques to profile employees’ well-being in the UK and France. They also find about five classes of employees. The major difference is that we extend the analysis to five European countries and look at a broader class of worries. Finally, instead of using latent class analysis, we use k-means clustering techniques to allocate workers among clusters.

Thirdly, we analyze and find that risk clusters are linked to employee socio-demographic characteristics, certain types of protective behaviors, and levels of social trust. Notably, a lack of trust in government actions stimulates economic and financial worries, while social trust limits many worries.

Fourth, the analysis is carried out and confirmed for five European countries, also recognizing that countries’ underlying social and economic fabrics influence concerns [18]. In particular, our analysis examines possible differences in social foundations along the lines of the work by [19]. Those country differences should and are confirmed to affect the relative size of clusters but not the clusters themselves by countries.

Finally, as the analysis is based on respondents’ self-reports, we use online response time to adjust survey responses based on the neuroeconomics principle that response time indicates attitude strength [20]. By correcting this response time, we refocus the responses towards a neutral response. This procedure thus reinforces our statistically significant results. This response adjustment procedure has also been used, among others, by [21]) in the context of assessing worries during covid-19, but with a focus on economic worries only.

## Materials and methods

This research is part of a vast multinational project on Covid-19, aimed at understanding people’s concerns and behaviors in the face of the pandemic. The focus is on the working population, which typically accounts for over 50% of all citizens and is a crucial driver of economic growth. We concentrated our efforts on the peak of the pandemic in April 2020 in European countries.

### Sample scope

Five countries were analyzed: France, Germany, Italy, Spain, and Sweden. These countries, the largest in Europe, represent different socio-economic models [19]. Additionally, these countries had varied policy responses to the Covid-19 crisis, with Sweden opting not to impose containment measures unlike the strict policies in place in Italy, for example. Researchers who wish to access the data can contact the NEURHOM company at at anna.szydlo@neurohm.com (Data officer of the company) and/or Rafal Ohme (CEO of the company) at rafal@neurohm.com. The company will facilitate the process according to the established confidentiality agreements. In addition, since this study involved an anonymous survey, and no personally identifiable information was collected, no ethical approval was required.

Data was collected online, based on representative country samples for age (over 18) and gender, and recruited via a panel agency in April 2020. The total sample comprised over 5,000 responses, or a minimum of 1,000 per country. Focusing on employees alone, the total sample consisted of just over 2,780 employees in five countries (Tables 1 and 2), or 55% of the sample, aligning with employee participation in the 18+ population of these countries.

**Table 1:** Number of respondents (employees) and demographic breakdown by country.

	Total	Type		Age			Total number of employees
	N	Women	Men	18-35	36-49	50+	N
FRANCE	1,024	51%	49%	29%	28%	43%	639
GERMANY	1,017	49%	51%	27%	24%	50%	535
ITALY	1,021	51%	49%	26%	30%	44%	507
SPAIN	1,019	50%	50%	32%	32%	36%	635
SWEDEN	1,006	51%	49%	30%	20%	49%	466

**Table 2:** High-level employee demographics and exposure to Covid.

Features	The types	Percentage	Features	The types	Percentage
Type	Woman	47%	Location	<100,000 inhabitants.	56%
	Men	53%		>100,000 inhabitants.	44%
			Income	<20,000€	29%
Age	<18	0%		>20,000€	71%
	18-25	7%		Do not wish to answer	7%
	26-35	23%	Infected	Yes	26%
	36-49	37%		No	68%
	50-64	31%		Don’t know	6%
	>64	2%		Do not wish to answer	1%
Education	Elementary school	2%	Political orientation	Left	23%
	College	8%		Law	26%
	Professional	28%		Other	21%
	Lycée	26%		Don’t get involved in politics	21%
	Baccalaureate or higher	35%		Do not wish to answer	8%
The children	0 children	50%			
	1 child	25%			
	2 children	19%			
	3 children	4%			
	>3 children	1%			

### Sample collection

Respondents were invited by email and informed about the scope of the study. Their task was to assess whether they agreed with the statements displayed on the screen. To avoid coercing responses or receiving answers that did not reflect actual behavior, each question was structured so that answers could be given on a three-point scale (yes, hard to say, no), with the “hard to say” option allowing respondents the flexibility to avoid forced choices.

We checked for the usual response biases, but the uncertainty between what people report and their actual attitudes/behaviors remains a significant caveat. We measure response time and adjust the data in line with [20], who finds a strong correlation between statements and actual behavior in individuals with fast reaction times when expressing their opinions. Similar to [21], the iCode Smart test was used to collect data [22], with response time (RT) collected for each response. Responses with a latency of less than 500 milliseconds (ms) (suspected of being given randomly) or greater than 10,000 ms (suspected of having been given after distraction) were eliminated. This accounted for less than 1% of doubtful responses.

To account for individual differences in reaction speed, we normalized the reaction time data measured in milliseconds. STDRT is the z-score of log(RT), with mean =  0 and standard deviation =  1. We then created a variable, RTC, that takes into account both the explicit response and the reaction time (RT) required to produce the response, i.e., RTC’ - 1/2 =  (1 - a) x (Y - N)/4 (0 < RTC’ <  1) where (1 - a) =  max(SDRT, 2)/2 and Y-N is the difference between the share of Yes and No reported. Thus, 0 <  a <  1 acts as a factor that reduces the difference between responses as a function of response reaction time, which we call the confidence index. When RTC’ converges towards 50%, it implies that everyone’s answer hovers around “Difficult to say,” or that not all answers are credible due to unusual reaction times. The more extreme the value of RTC’, the firmer the position taken by the survey response on the qualification of the statement asked for in the survey. Thus, RTC =  0 corresponds to a solid and dominant NO, and RTC =  1 to an overwhelming YES.

### High-level statistics

#### Worries.

We consider worries as a natural precursor of well-being, as argued by [23]. We examine four types of concern: health (H), economic (E), social (S), and psychological (P). These concerns align with other studies that extend beyond purely health issues related to the pandemic [13,14]. Each worry domain is based on four dimensions, representing different intensities or scopes, e.g., the health concept concerns the self, close ties (children and elderly members), and weak ties; social worries affect family tensions, escalating to domestic violence or divorce. Table 3 shows the RTC value and the confidence index of the responses associated with each risk or concern, ranked from most important to least important, for the entire sample and for the 16 dimensions that comprise H, E, S, and P.

**Table 3:** Concern of European employees during wave 1 of the Covid-19 pandemic.

RTC	Trust	Declaration
0.70	0.43	I worry about the health of elderly family members (H)
0.67	0.41	I’m concerned about the health of people in my country (H)
0.63	0.49	COVID-19 increases domestic violence (S)
0.62	0.61	The COVID-19 epidemic will make society more unequal (S)
0.60	0.53	I’m afraid our country is running out of money (E)
0.60	0.49	I’m worried I won’t be able to meet my family (P)
0.59	0.52	COVID-19 will increase divorce rates (S)
0.57	0.52	I’m anxious about not being able to meet my friends (P)
0.54	0.58	Living in isolation has a negative impact on my well-being (P)
0.54	0.31	I’m worried about my health (H)
0.54	0.35	I worry about my children’s health (H)
0.54	0.63	Being together all the time increases family tensions (S)
0.53	0.59	I worry about the effect isolation will have on me (P)
0.52	0.35	I’m worried about my financial situation (E)
0.47	0.36	I’m worried about my professional situation (E)
0.44	0.59	I’m afraid there aren’t enough necessities in the stores (E)

Adding up all the values, the total is 9.1 out of 16, a value of 56.8%. This indicates that most concerns prevail in the employee population during the first wave of the Covid-19 pandemic. Furthermore, H =  61.2%, S = 59.5%, P =  56.0% and E =  50.7%, meaning that each factor is predominantly present in the employee population. Finally, H has the highest value but represents only 40% of the total concerns expressed. The main H concern relates to people at increased risk of death, such as elderly family members, consistent with the findings of other publications, such as [24].

The economic dimensions give rise to different concerns. Supply chain risk (measured by the availability of needed goods) is the least important concern, while the macroeconomic risk of a cash-strapped country is a broader concern. Psychological/mental and social risks are also significant, and domestic violence and divorce rates are risky for the employees sampled.

As our sample selects only employees, we can also compare the magnitude of concern with that of non-salaried employees, e.g., retirees or people of working age who are not working. In this case, the total for retirees is 7.8 (15% lower than for salaried employees), while 8.7 (6% lower than for salaried employees) for the other non-working population. In other words, the salaried population expresses a broader range of concerns than the non-salaried. The weaker expression of worries is explained in particular by economic considerations, with the most significant difference between retirees and salaried employees being by far the risk of job loss [25]. Not surprisingly, retirees are more concerned about their health and less about social risks (as they tend to be more autonomous).

**Fig 1:**
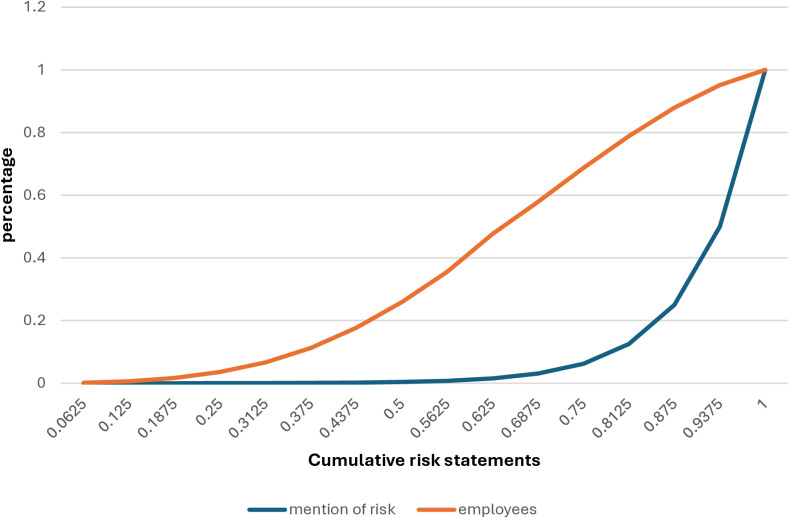
Distribution of Covid-19 risk expression among employees.

Fig 1 also shows the distribution of risk expression among employees is far from uniform. We calculate that 20% of employees express less than 50% of the type of H, S, P, E risks studied, and 20% of others mention at least 81% during the Covid-19 pandemic. In addition, 18% of employees account for 90% of all concerns or are more concentrated than a typical Pareto distribution.

This type of “power law” is observed in many phenomena. In economics, power laws can be seen in income and wealth, city and company size, stock market returns, or international trade [26]. The power law has also recently been studied as the mechanism underlying the spread of the covid-19 pandemic around the world [27]. The power law has also been studied in the context of psychological disorders, e.g., [28], but this is the first time, to our knowledge, that it is examined in the context of general well-being affected by a significant disturbance such as a pandemic.

In particular, our study focuses on two essential aspects of this apparent power law: how the distribution is determined by the variety and intensity of worries and how perceptions/attitudes correlate with worries.

#### Contextual factors.

Worries are known to be correlated with sociodemographics and lifestyle, for instance, older people tend to worry more about health issues given the high risk linked to contamination, etc. [29]. We also have collected responses related to various attitudes/behaviors and trusts likely to correlate employees’ expression of risk. As we had a long list of statements, we first applied categorical principal component analysis (CATPCA) to reduce the information. CATPCA was performed using Varimax rotation with Kaiser normalization to maximize the sum variance of the factor coefficients.

Ten factors were derived, representing 69.6% of the total variance. Table 4 presents the ten factors and associated dimensions in the order in which they emerged from the data rotation. Table 5 shows the RTC’ and confidence values, ranked in order of importance of the factors.

**Table 4:** PCA factors from European employees’ declarations related to the Covid-19 pandemic.

Factor	Dimensions	Dimensional loading
1. Trust in institutions	I am satisfied with the way my government is handling this crisis.	0.912
	The government is doing a good job on COVID-19	0.908
	Government publishes actual figures for coronavirus infections and deaths	0.702
	The [CHAIRMAN] is doing a good job on COVID-19.	0.608
	The media provide reliable information on the pandemic	0.519
2. NPI compliance	I respect the physical distance recommendations	0.683
	I respect the restrictions that require me to stay at home	0.619
	I wash my hands for 20 seconds if necessary	0.600
	I’m grateful to our healthcare professionals	0.594
	I actively encourage others to respect restrictions and guidelines.	0.549
3. Social fabric/citizenship	Since COVID-19, I exercise more at home	0.607
	Since COVID-19, I’ve been eating healthier	0.603
	I’m worried about my children’s education	0.561
	I’d like to help the most vulnerable at COVID-19	0.534
	COVID-19 will bring countries closer together	0.482
	I fear an increase in burglaries and thefts.	0.435
4. Confidence in healthcare	In the event of coronavirus infection, I will receive the appropriate medical assistance.	0.711
	I am grateful to our essential workers	0.599
	I’m pleased with the way our healthcare system is handling this crisis.	0.590
	I think we’ll beat COVID-19 soon.	0.304
5. Additional precautions	I disinfect the groceries before putting them away.	0.902
	I disinfect mail and deliveries before opening them.	0.899
6. Trust in people	COVID-19 reveals the worst in humans	−0.788
	COVID-19 brings out the best in people	0.775
	People will soon stop following restrictions	−0.571
7. Lifestyle maintenance	Since COVID-19, I’ve been exercising less	−0.745
	Since COVID-19, I’ve been eating less healthily	−0.740
8. Vulnerability	I have a high probability of contracting COVID-19.	0.839
	The coronavirus is dangerous for my health	0.663
9. Covid, a top priority	The media exaggerate the situation with COVID-19	−0.845
	Slowing the spread of COVID-19 is more important than saving money	0.434
	When the COVID-19 vaccine becomes available, I’d like to be vaccinated.	0.375
10. Covid duration	Restrictions caused by COVID-19 will be lifted within a month	−0.807
	Restrictions caused by COVID-19 will continue until at least autumn.	0.795

Notes: Main variable normalization. Rotation method: Varimax with Kaiser normalization.

**Table 5:** How do employees perceive the Covid-19 pandemic and act accordingly.

RTC	Trust	Factors
0.72	0.44	NPI compliance
0.66	0.52	Confidence in healthcare
0.57	0.51	Lifestyle maintenance
0.56	0.47	Vulnerability
0.55	0.54	Trust in people
0.54	0.50	Confidence in government
0.53	0.58	Covid, a top priority
0.52	0.52	Social fabric
0.44	0.49	NPI Additional precaution
0.41	0.67	Duration of crisis

Three factors (1, 4, 6) are linked to third-party trust. The first relates to government institutions, the second to healthcare, and the last to how people react to the Covid-19 crisis. Factors 2 and 5 relate to precautionary measures (NPI), with factor 2 encompassing the most critical ones in disease control [30]. Factors 8, 9, and 10 are all linked to the perception of a lasting danger from the virus. Factor 10 is linked to the duration of the crisis, and factors 8 and 9 to vulnerability to the virus and the priority given to health over wealth. Finally, factors 3 and 7 are more closely linked to social support for the individual and the family.

Grouped by themes, Table 4 shows good compliance with the NPI (mean =  62%), even after correcting for probably overestimated answers. The confidence level is the lowest of all themes. This overestimation could be explained by the fact that responses seem to obey the public mandate of quarantine and social distancing measures to limit the spread of pandemics. Nevertheless, as many studies have shown, RTC’ <  100%, i.e., NPI compliance, is incomplete [31]. Third-party trust is relatively well recognized (58%), but people feel very vulnerable (56%). The impact on lifestyle is felt to be more minor (48%). Expectations for the duration of the crisis are that it could be more short-term than long-term, so it seems that most European employees were not necessarily expecting the current second wave.

Trust is significantly higher in healthcare than in government. Nevertheless, public authorities should be trusted to manage the crisis so that citizens adopt the recommended protective actions [18,32]. Finally, vulnerability is more or less perceived by the employee population, and generally speaking, there is a majority to think the crisis will last.

#### Methodology.

We use a two-step process to analyze the distribution of worries and mediation factors. First, we perform clustering analysis to determine worry segments, then launch logit regression analysis to correlate factors associated with segments.

We prefer a two-step process instead of a complete clustering of all variables in one step, as mediation factors are not at the same level as worry expression. Further, the second step allows us to statistically assess the strengths of mediation factors, while direct mix clustering would prevent this information.

Regarding clustering, we use K-means clustering around the 16 items that present the four areas of concern H, S, P, E, to divide the population into coherent and stable segments. The study by [33] segments risk attitudes via K-means for Bangladesh. However, the study only involves 340 people online and given the country’s current digital development, is not representative of the population. Finally, drivers of group membership factors are not tested, as they are in this study.

The K-means technique minimizes the sum of the squared distances between each possible risk cluster and its centroid and allocates each employee to one cluster. Alternative clustering solutions exist, of which latent class analysis can be possible, as done, e.g., in [17]. The strength of the K-means is that it always ensures that the most similar observations are in the same clusters and that each observation is univocally allocated to one segment versus latent class analysis. Extra analyses with different cluster solutions were carried out, but the 5-cluster solution appears to be the most informative.

## Results and discussion

Table 6 shows segment size overall, then by country. We can see that the size of a segment depends on the country. Segment 5 is the largest in Sweden and Germany but the smallest in Italy and Spain. As we shall see later, segment five is made up of the least worried employees, unlike the first segment. The fact that Italy and Spain have such large worried workforces can be explained by the fact that these countries have suffered relatively high contamination, under-capacity in healthcare, and largely imposed containment. This contrasts with Sweden, where no containment was applied, or Germany, where healthcare capacity is relatively high and contamination was less widespread than in southern Europe.

**Table 6:** K-means cluster sizes of European employees for different risks associated with the Covid-19 pandemic.

Group of companies	Total	Germany	Spain	France	Italy	Sweden
1	30.4%	19.8%	46.5%	32.1%	31.2%	17.8%
2	15.6%	10.7%	18.6%	13.8%	23.1%	11.8%
3	15.4%	21.1%	9.4%	13.9%	16.8%	17.4%
4	21.2%	23.0%	20.8%	25.8%	15.8%	19.5%
5	17.3%	25.4%	4.7%	14.4%	13.2%	33.5%
Total	100%	100%	100%	100%	100%	100%

**Fig 2:**
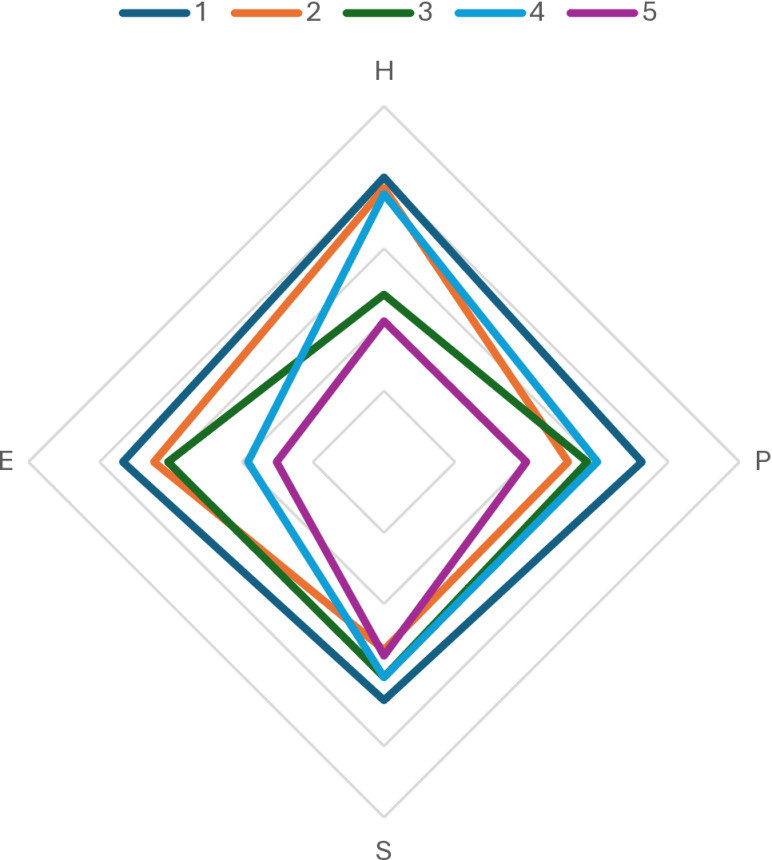
Covid-19 Radar risk profile by segment, European population.

Table 7 shows the mean values per segment for the 16 risks analyzed, while Fig 2 groups the risk profiles according to the four domains H, P, S, E. We confirm that the segments concerned vary in intensity and variety of the risks expressed.

**Table 7:** Expression of Covid-19 pandemic-related risks by European employee segment.

Segment	1	2	3	4	5
I’m worried about my financial situation	80%	77%	74%	28%	22%
I’m worried about my professional situation	79%	73%	66%	22%	20%
I’m afraid our country is running out of money.	73%	66%	62%	61%	46%
I’m afraid there aren’t enough basic necessities in the stores.	62%	43%	42%	42%	33%
I’m worried about my health	84%	82%	30%	74%	22%
I’m worried about my children’s health	77%	71%	37%	69%	30%
I’m concerned about the health of elderly family members	79%	78%	63%	79%	57%
I’m worried about the health of the people of my country	80%	77%	59%	80%	49%
I’m anxious about not being able to meet my friends.	71%	50%	59%	63%	41%
I’m worried I won’t be able to meet my family.	77%	60%	58%	69%	40%
I worry about the effect isolation will have on me	73%	50%	53%	52%	37%
Living in isolation has a negative impact on my well-being	69%	48%	58%	56%	41%
The COVID-19 epidemic will make society more unequal	68%	60%	63%	64%	56%
Being together all the time increases family tensions	65%	47%	54%	53%	47%
COVID-19 increases domestic violence	67%	51%	65%	65%	61%
COVID-19 will increase divorce rates	68%	53%	59%	61%	54%
Total risk mentions out of 16	12.91	8.79	7.78	8.62	4.26

For simplicity, we name the cluster rank according to the decreasing intensity of the level of concern expression - i.e., the greatest for the first segment.

**Fig 3:**
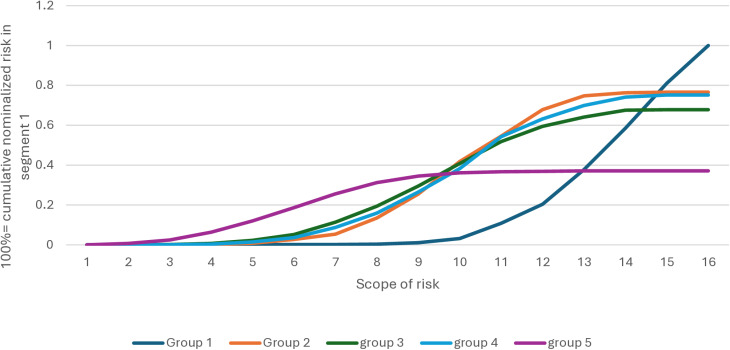
Distribution of risk mentions by population segment.

Segment 5 is the only segment whose average RTC’ is <  50% (Fig 3), except that segment 5 perceives social risk similarly to the average of the other segments. Its primary concern is social and is linked to domestic violence and divorce rates.

Table 8 describes compliance with essential non-pharmaceutical interventions (NPIs) as a check to cluster validity. We see that greater use of NPIs is linked to more anxious segments [34–36]. In particular, segment 5 is less compliant with any measure, as it suffers from the lowest level of risk. Segments 1 and 2 prefer to be quarantined or choose to have as few interactions as possible, as they bear the most considerable weight of risk. Segment 2 conforms best to midlife, as it suffers relatively less from loneliness. Segment 3 is relatively unconcerned about its health and more concerned about preserving its job, so it conforms more to social distancing than midlife.

**Table 8:** NPI compliance by European employees, by risk segment.

Segment	1	2	3	4	5
NPI	77%	78%	70%	76%	68%
I respect the physical distance recommendations	77%	78%	72%	78%	72%
I respect the restrictions that require me to stay at home	79%	81%	68%	76%	64%
I wash my hands for 20 seconds if necessary	76%	75%	69%	75%	69%

In addition to NPI compliance being a good marker of segment concerns, we now formally test the existence of multivariate segmentation markers using a formal logit statistical model. We include socio-economic factors, as they are well known to have an impact on attitudes and the expression of concerns (e.g., [24,37], and to condition the ability to work remotely [10]).

Each segment logit regression against Segment 1 is presented in Appendix 2. At the same time, Table 9 summarizes the results, showing only those markers that are statistically significant at the 10% statistical threshold and omitting the country effect. For the sake of simplicity, we have also grouped the factors into four broad categories (confidence, NPI compliance, vulnerability and lifestyle). A negative sign means a weaker impact on the probability of belonging to a segment.

**Table 9:** Probit estimates of risk segment membership.

Segment	1		2		3		4		5	
	coeff.	s.e.	coeff.	s.e.	coeff.	s.e.	coeff.	s.e.	coeff.	s.e.
1. confidence in institutions	−0.2	0.082	0.15	0.09			−0.24	0.08	0.45	0.1
4. Confidence in healthcare			−0.4	0.13	−0.33	0.13	0.67	0.13		
6. Trust in people	−0.42	0.123	0.24	0.13					0.26	0.15
2. NPI compliance	0.7	0.123	0.64	0.16	−0.46	0.12	0.51	0.13	−0.63	0.14
5. Additional precautions	0.15	0.058	0.28	0.06	−0.27	0.07	−0.17	0.06	−0.16	0.08
8. Vulnerability	0.72	0.123	0.51	0.09	−0.72	0.08	0.37	0.07	−0.91	0.09
9. Covid, a top priority	0.46	0.067	−0.5	0.13						
10. Covid duration										
Social fabric/citizenship							−0.31	0.11	−1.77	0.16
Lifestyle maintenance				0.07	0.16	0.07	−0.15	0.07	−0.48	0.09
Elementary school	−0.74	0.442	0.85	0.38						
College			0.38	0.21						
Professional									−0.26	0.16
Lycée			0.24	0.14						
< 100,000 inhabitants					−0.29	0.12				
< 2,000 euros/month									−0.53	0.26

Socio-demographic data play a specific role in predicting group membership, but not a major one. Neither family composition nor gender has a statistically significant impact.

A low income (less than 2,000 euros per month) reduces the probability of belonging to segment 5. One of the reasons, already highlighted in the introduction to this study, is that low income is often associated with essential on-site work, exposing people to health risks and vulnerability to the virus. Living in the countryside (in towns with fewer than 100,000 inhabitants) makes an employee less likely to belong to segment 3. The level of education affects the probability of belonging to different segments.

The markers of trust, vulnerability and lifestyle play a significant role in the distribution of employees between the different groups. Perceived vulnerability is an essential discriminating factor in all segments, favoring health risk. Compliance with the NPI (and increased caution) are behaviors that emerge from health risk, but we find that they play a role in addition to vulnerability. One reason for this is that the NPI was imposed as a response to a government mandate and reflects compliance with authoritative measures.

Trust is also essential. Segments 1 and 3 are less inclined to accept their government’s actions to combat the Covid-19 pandemic. Segment 1 is also a segment that trusts its peers more than institutions, for example.

Using the exponential of the point estimates in Table 9, Table 10 calculates the marginal probability impact for the four marker categories. Consider an employee among others with low institutional trust, who also complies with the NPI and feels vulnerable to the virus, who belongs to segment 1. Its counterpart belongs to segment 5. These two segments are also the most and least fragile among employees. An employee unafraid of the virus trusts the healthcare system, respects the NPI while maintaining a healthy lifestyle, and most likely belongs to segment 4.

**Table 10:** How the markers determine the risk segments associated with Covid-19.

Segment	1	2	3	4	5
Trust	−18%	4%	−9%	25%	29%
NPI	59%	61%	−30%	26%	−31%
Dangerous viruses	55%	9%	−17%	15%	−20%
Lifestyle	0%	48%	66%	53%	−7%
Total	96%	122%	10%	118%	−29%

While segment 5 is less “fragile”, it may represent a risk for other segments, due to low NPI compliance. Similarly, human resources need to adopt a fine-tuned approach to support employees effectively: Segment 4 may agree with the health situation related to Covid, but is more stressed regarding employment. Segment 1 and, to a lesser extent, segment 2 are rather stressed and have physical and mental health issues to resolve if they are to remain productive.

A final remark concerns the differences between countries. Table 11 illustrates the impact of markers on the first segment in the five countries concerned. As with the total, greater compliance with the NPI, greater perception of Covid-19’s vulnerability, strong social fabric or lifestyle maintenance are the norm in all countries.

**Table 11:** Marginal probability of belonging to the most fragile segment (segment 1).

	Sweden	Germany	France	Spain	Italy
Confidence in government				−33%	
Confidence in healthcare					
Trust in people				95%	
NPI compliance	369%	191%	208%	132%	252%
Additional precautions					
Vulnerability	247%	300%	185%	155%	278%
Top priority	0%	252%	0%	87%	136%
Duration of agreement					
Social fabric	753%	278%	397%	480%	272%
Lifestyle maintenance	116%	99%	107%	116%	108%

Note: only coefficients statistically significant at 10% are included.

Spanish employees are more sensitive to trust in people than in government, while Swedish employees are better predicted by their NPI compliance level and social fabric. Regarding social priorities, German employees are more sensitive to health than wealth. Again, these tendencies can be attributed to culture and background, and Sweden has a robust social culture compared to other countries [19] and has not imposed confinement. Swedish employees’ compliance with the NPI is likely to be a more evident discriminatory behavior than in countries where the NPI has been set.

## Conclusions

In the context of a major viral pandemic, employee well-being should be a central concern. Employees can face a significant risk of contagion while working on-site and represent one of the primary resources of an economy already strained by the major disruptions induced by the pandemic.

We find that well-being can be severely affected, as employees express a wide variety of concerns beyond physical health, including mental stress and job preservation concerns which, if left unaddressed, can seriously undermine productivity. These concerns may persist even if people telework; other stresses, such as domestic violence, may emerge.

More importantly, we show that workforce fragility is not evenly distributed and exhibits a kind of power curve whereby nearly 20% of employees are responsible for 90% of the variety and intensity of worries. We find that five well-defined segments can be identified using clustering techniques to represent this power law. In addition, we can associate a set of critical markers that predict employee worries. These markers may be particularly useful, for example, for humane enterprises to engage in appropriate selective dialogue with their employees and improve their well-being during this pandemic. This is not just a matter of corporate responsibility but an effective strategy for maintaining high productivity and ensuring business resilience.

This study, while comprehensive, has several limitations. Firstly, the data collection was conducted online and is based on self-reports, which may introduce response biases. Participants might underreport or overreport their stress levels and concerns due to social desirability or other psychological factors. Additionally, the study relies on respondents’ ability to accurately recall and assess their experiences during the pandemic, which may be influenced by memory biases.

Secondly, the study focuses on a specific period during the acute phase of the Covid-19 pandemic. As the situation evolves, the stressors and concerns of employees may change, potentially altering the relevance and accuracy of the identified clusters and their characteristics. Longitudinal studies are necessary to capture these dynamic changes and provide a more comprehensive understanding of employee well-being over time.

Thirdly, the sample is limited to five European countries, which, while diverse, may not fully represent the global workforce’s experiences. Cultural, economic, and social differences across regions could influence the generalizability of the findings. Future research should include a more varied international sample to validate and expand upon these results.

To address these limitations, several remedies can be considered. To mitigate response biases, future studies could incorporate mixed methods, including qualitative interviews and focus groups, to complement quantitative surveys and provide a deeper understanding of employee experiences. Additionally, employing objective measures of stress, such as physiological indicators, could help validate self-reported data.

Longitudinal studies should be conducted to track changes in employee well-being over time, capturing the evolving nature of stressors and concerns as the pandemic progresses or resolves. This approach will provide a more nuanced understanding of how employees adapt and what interventions are most effective at different stages.

Expanding the sample to include a broader range of countries and regions will enhance the generalizability of the findings. Cross-cultural comparisons can reveal how different socio-economic contexts and government responses impact employee well-being, offering insights that are globally applicable.

## Appendix 1

### Tested statements

**Table:** 

Behavior
1. I actively encourage others to follow the restrictions and guidelines
2. I comply with the recommendations for physical distancing
3. I comply with the restrictions to stay home
4. I disinfect groceries before putting them away
5. I disinfect mail and deliveries before opening them
6. I wash hands for 20 seconds when necessary
7. I would like to help people who are more vulnerable to COVID-19
8. Since COVID-19 I eat healthier
9. Since COVID-19 I eat unhealthier
10. Since COVID-19 I exercise less
11. Since COVID-19 I exercise at home more
12. When a COVID-19 vaccine is available, I’d like to be vaccinated
**Emotions**
13. I’m worried about my financial situation
14. I’m worried about my job situation
15. I’m worried that our country will run out of money
16. I’m worried that there will not be enough basic necessities in the stores
17. I am worried about my own health
18. I am worried about the health of my children
19. I am worried about the health of my older family members
20. I am worried about the health of people in my country
21. I worry that there will be an increase in break-ins and thefts
22. I’m worried about my children’s education
23. I am anxious about not being able to meet with friends
24. I am worried about not being able to meet with my family
25. I worry how living in isolation will affect me
26. Living in isolation negatively impacts my well-being
**Opinions**
27. The COVID-19 outbreak will make society more unequal
28. Being together all the time increases family tensions
29. COVID-19 increases domestic violence
30. COVID-19 will increase divorce rates
31. COVID-19 will bring countries closer
32. I am grateful to our essential workers
33. I am grateful to our healthcare professionals
34. My chance of getting COVID-19 is high
35. Slowing the spread of COVID-19 is more important than the economy
36. Coronavirus is dangerous for my health
37. Media exaggerate the situation with COVID-19
38. Media provide reliable information about the pandemic
39. [The President] is doing a good job dealing with COVID-19
40. I am satisfied with how my government is handling this crisis
41. The government is doing a good job dealing with COVID-19
42. I am satisfied with how our healthcare system is handling this crisis
43. In the case of coronavirus infection, I will get appropriate medical help
44. The government discloses real numbers of coronavirus infections and deaths
45. COVID-19 reveals the best in people
46. COVID-19 reveals the worse in people
47. I believe we will beat COVID-19 soon
48. People will stop following the restrictions soon
49. The restrictions caused by COVID-19 will continue at least until the fall
50. The restrictions caused by COVID-19 will continue for about a month

## Appendix 2

### Probit estimates

**Table:** 

CLUSTER 1 [K-Means 5 clusters for risk perception RTC]^a^		B	Std. Error	Wald	df	Sig.	Exp(B)	95% Confidence Interval for Exp(B)	
Lower Bound	Upper Bound
	Intercept	−2.478	0.203	149.006	1	0.000			
	[Gender - Male=1,00]	−0.223	0.107	4.364	1	0.037	0.800	0.649	0.986
	[Gender - Male=2,00]	0^b^			0				
	[Kids - 0 children=1,00]	−0.293	0.112	6.823	1	0.009	0.746	0.599	0.929
	[Kids - 0 children=2,00]	0^b^			0				
	Income - < 20000€ = 1,00]	0.303	0.117	6.664	1	0.010	1.354	1.076	1.704
	Income - < 20000€ = 2,00]	0^b^			0				
	[Quarantine - yes=1,00]	−0.366	0.176	4.339	1	0.037	0.693	0.491	0.979
	[Quarantine - yes=2,00]	0^b^			0				
	Factor02_RTC - Compliance	0.517	0.154	11.321	1	0.001	1.677	1.241	2.266
	Factor03_RTC - Social citizenship	1.428	0.126	129.322	1	0.000	4.171	3.261	5.335
	Factor05_RTC - Extra caution	0.166	0.060	7.560	1	0.006	1.180	1.049	1.328
	Factor06_RTC - Bad in people	0.585	0.120	23.933	1	0.000	1.795	1.420	2.269
	Factor07_RTC - Lifestyle impact	0.641	0.070	83.711	1	0.000	1.899	1.655	2.179
	Factor08_RTC - Percived vulnerability	0.570	0.083	46.882	1	0.000	1.768	1.502	2.081
	Factor09_RTC - Fighting Covid top priority	0.594	0.125	22.384	1	0.000	1.811	1.416	2.315
	Factor10_RTC - Predictions	0.298	0.121	6.087	1	0.014	1.347	1.063	1.708
	GAP_INF [mean Std-RT from 16 risk perception attributes]	−4.019	0.292	188.861	1	0.000	0.018	0.010	0.032

a The reference category is other clusters.

b This parameter is set to zero because it is redundant.

**Table:** 

CLUSTER 2 [K-Means 5 clusters for risk perception RTC]^a^		B	Std. Error	Wald	df	Sig.	Exp(B)	95% Confidence Interval for Exp(B)	
Lower Bound	Upper Bound
	Intercept	−2.170	0.185	137.545	1	0.000			
	[country_DE=1,00]	−0.462	0.171	7.261	1	0.007	0.630	0.450	0.882
	[country_DE=2,00]	0^b^			0				
	[country_IT=1,00]	0.474	0.137	12.067	1	0.001	1.607	1.230	2.100
	[country_IT=2,00]	0^b^			0				
	[Edu - Bachelor or higher=1,00]	−0.294	0.121	5.907	1	0.015	0.745	0.588	0.945
	[Edu - Bachelor or higher=2,00]	0^b^			0				
	[Infected - don’t want to answer=1,00]	−20.349	0.000		1		1.455E-09	1.455E-09	1.455E-09
	[Infected - don’t want to answer=2,00]	0^b^			0				
	Factor02_RTC - Compliance	0.628	0.151	17.220	1	0.000	1.874	1.393	2.522
	Factor04_RTC - Trust in healthcare	−0.426	0.121	12.470	1	0.000	0.653	0.516	0.827
	Factor05_RTC - Extra caution	0.327	0.060	29.954	1	0.000	1.387	1.234	1.560
	Factor06_RTC - Bad in people	−0.270	0.119	5.147	1	0.023	0.763	0.604	0.964
	Factor07_RTC - Lifestyle impact	−0.330	0.076	19.008	1	0.000	0.719	0.620	0.834
	Factor08_RTC - Percived vulnerability	0.585	0.091	41.352	1	0.000	1.794	1.502	2.144
	Factor09_RTC - Fighting Covid top priority	−0.449	0.129	12.041	1	0.001	0.638	0.495	0.823
	GAP_INF [mean Std-RT from 16 risk perception attributes]	1.869	0.273	46.747	1	0.000	6.484	3.794	11.081

a The reference category is other clusters.

b This parameter is set to zero because it is redundant.

**Table:** 

CLUSTER 3 [K-Means 5 clusters for risk perception RTC]^a^		B	Std. Error	Wald	df	Sig.	Exp(B)	95% Confidence Interval for Exp(B)	
Lower Bound	Upper Bound
	Intercept	−1.414	0.157	80.696	1	0.000			
	[Gender - Female=1,00]	0.277	0.116	5.672	1	0.017	1.319	1.050	1.656
	[Gender - Female=2,00]	0^b^			0				
	Age - 50-64 = 1,00]	−0.313	0.133	5.505	1	0.019	0.731	0.563	0.950
	Age - 50-64 = 2,00]	0^b^			0				
	[Kids - 0 children=1,00]	0.458	0.118	15.050	1	0.000	1.581	1.254	1.993
	[Kids - 0 children=2,00]	0^b^			0				
	[Town ->100000 inhab. = 1,00]	0.275	0.116	5.665	1	0.017	1.317	1.050	1.652
	[Town ->100000 inhab. = 2,00]	0^b^			0				
	Income - < 20000€ = 1,00]	0.270	0.127	4.568	1	0.033	1.311	1.023	1.679
	Income - < 20000€ = 2,00]	0^b^			0				
	Factor02_RTC - Compliance	−0.426	0.118	13.018	1	0.000	0.653	0.518	0.823
	Factor04_RTC - Trust in healthcare	−0.544	0.119	21.037	1	0.000	0.580	0.460	0.732
	Factor05_RTC - Extra caution	−0.246	0.066	14.087	1	0.000	0.782	0.688	0.889
	Factor07_RTC - Lifestyle impact	0.182	0.073	6.233	1	0.013	1.200	1.040	1.385
	Factor08_RTC - Percived vulnerability	−0.759	0.079	91.549	1	0.000	0.468	0.401	0.547
	GAP_INF [mean Std-RT from 16 risk perception attributes]	1.617	0.272	35.204	1	0.000	5.037	2.953	8.592

a The reference category is other clusters.

b This parameter is set to zero because it is redundant.

**Table:** 

CLUSTER 4 [K-Means 5 clusters for risk perception RTC]^a^		B	Std. Error	Wald	df	Sig.	Exp(B)	95% Confidence Interval for Exp(B)	
Lower Bound	Upper Bound
	Intercept	−2.262	0.168	181.827	1	0.000			
	[country_IT=1,00]	−0.504	0.142	12.616	1	0.000	0.604	0.458	0.798
	[country_IT=2,00]	0^b^			0				
	[country_SE=1,00]	−0.401	0.141	8.102	1	0.004	0.670	0.508	0.883
	[country_SE=2,00]	0^b^			0				
	[Kids - 2 children=1,00]	0.254	0.119	4.580	1	0.032	1.289	1.022	1.627
	[Kids - 2 children=2,00]	0^b^			0				
	[Town - <100000 inhab.=1,00]	0.221	0.099	4.921	1	0.027	1.247	1.026	1.515
	[Town - <100000 inhab.=2,00]	0^b^			0				
	Income - < 20000€ = 1,00]	−0.341	0.115	8.840	1	0.003	0.711	0.568	0.890
	Income - < 20000€ = 2,00]	0^b^			0				
	Factor02_RTC - Compliance	0.684	0.130	27.704	1	0.000	1.981	1.536	2.555
	Factor03_RTC - Social citizenship	−0.410	0.107	14.754	1	0.000	0.664	0.539	0.818
	Factor04_RTC - Trust in healthcare	0.363	0.113	10.322	1	0.001	1.437	1.152	1.793
	Factor05_RTC - Extra caution	−0.189	0.058	10.460	1	0.001	0.828	0.738	0.928
	Factor07_RTC - Lifestyle impact	−0.146	0.063	5.344	1	0.021	0.864	0.763	0.978
	Factor08_RTC - Percived vulnerability	0.399	0.074	29.431	1	0.000	1.491	1.291	1.722
	GAP_INF [mean Std-RT from 16 risk perception attributes]	0.499	0.237	4.410	1	0.036	1.646	1.034	2.622

a The reference category is other clusters.

b This parameter is set to zero because it is redundant.

**Table:** 

CLUSTER 5 [K-Means 5 clusters for risk perception RTC]^a^		B	Std. Error	Wald	df	Sig.	Exp(B)	95% Confidence Interval for Exp(B)	
Lower Bound	Upper Bound
	Intercept	−0.912	0.144	40.127	1	0.000			
	[country_ES=1,00]	−0.622	0.222	7.876	1	0.005	0.537	0.347	0.829
	[country_ES=2,00]	0^b^			0				
	[country_SE=1,00]	0.537	0.147	13.251	1	0.000	1.710	1.281	2.283
	[country_SE=2,00]	0^b^			0				
	[Gender - Female=1,00]	−0.458	0.126	13.175	1	0.000	0.633	0.494	0.810
	[Gender - Female=2,00]	0^b^			0				
	Income - < 20000€ = 1,00]	−0.391	0.149	6.880	1	0.009	0.676	0.505	0.906
	Income - < 20000€ = 2,00]	0^b^			0				
	Factor01_RTC - Trust in Government	0.394	0.087	20.280	1	0.000	1.483	1.249	1.760
	Factor02_RTC - Compliance	−0.555	0.127	19.069	1	0.000	0.574	0.448	0.737
	Factor03_RTC - Social citizenship	−1.822	0.144	161.056	1	0.000	0.162	0.122	0.214
	Factor06_RTC - Bad in people	−0.326	0.134	5.901	1	0.015	0.722	0.555	0.939
	Factor07_RTC - Lifestyle impact	−0.456	0.084	29.378	1	0.000	0.634	0.537	0.747
	Factor08_RTC - Percived vulnerability	−0.865	0.085	103.806	1	0.000	0.421	0.356	0.497
	GAP_INF [mean Std-RT from 16 risk perception attributes]	1.119	0.305	13.458	1	0.000	3.063	1.684	5.570

a The reference category is other clusters.

b This parameter is set to zero because it is redundant.
